# Cartilage Organoids from Articular Chondroprogenitor Cells and Their Potential to Produce Neo-Hyaline Cartilage

**DOI:** 10.1177/19476035241313179

**Published:** 2025-02-10

**Authors:** Daphne M.A. Menssen, Jeske C.A. Feenstra, Rob P.A. Janssen, Florencia Abinzano, Keita Ito

**Affiliations:** 1 Orthopaedic Biomechanics, Department of Biomedical Engineering, and Institute for Complex Molecular Systems, Eindhoven University of Technology, Eindhoven, The Netherlands; 2 Department of Orthopaedic Surgery and Trauma, Máxima Medical Center Eindhoven-Veldhoven, Eindhoven, The Netherlands; 3 Department of Paramedical Sciences, Fontys University of Applied Sciences, Eindhoven, The Netherlands

**Keywords:** cartilage repair, organoids, chondrocytes, articular chondroprogenitor cells, tissue engineering

## Abstract

**Introduction:**

The use of autologous human primary articular chondrocytes (hPACs) for repairing damaged cartilage is the golden standard; however, their 2-dimensional (2D) expansion induces dedifferentiation, making it challenging to create hyaline cartilage. Spinner flasks are efficient for generating cartilage organoids, allowing hPACs to proliferate without dedifferentiation; however, porcine notochordal cell-derived matrix (NCM) is needed for aggregation, limiting clinical application. Human articular chondroprogenitor cells (hACPCs) can be expanded many fold while maintaining chondrogenic potential. Therefore, the scalable production of hACPC cartilage organoids without NCM in spinner flasks was investigated in this study.

**Methods:**

hPAC organoids with NCM and hACPC organoids using bone morphogenetic protein 9 (BMP-9) were produced in spinner flasks in 14 days. Thereafter, approximately 20 organoids were fused in low adhesive wells for 21 days. Organoids underwent mechanical testing, and both organoids and fused constructs were evaluated using biochemical, histological, and immunohistochemical analysis.

**Results:**

The hACPCs self-assembled and synthesized abundant extracellular matrix once stimulated with BMP-9. The hPAC and hACPC organoids showed similar mechanical properties, but hACPC organoids and fused constructs showed a more uniform matrix and cell distribution.

**Conclusion:**

The hACPC organoids fused into a neo-hyaline cartilage-like tissue, demonstrating their potential for improved, scalable cartilage tissue repair.

## Introduction

Articular cartilage provides a lubricated surface in joints facilitating smooth and low-friction movement, as well as the distribution of loads. The tissue consists mostly of water and a dense extracellular matrix (ECM) predominantly composed of glycosaminoglycans (GAGs) and type II collagen. Articular cartilage only contains a few cells called chondrocytes.^1^ Within the ECM, chondrocytes are encapsulated within a softer pericellular matrix (PCM), composed of type VI collagen and perlecan.^2^ Furthermore, cartilage is avascular, aneural, and alymphatic, resulting in a low regenerative potential.^3^ Therefore, damaged cartilage often requires treatment to relieve symptoms and prevent progression toward osteoarthritis.^3^ Multiple treatments for cartilage defects are available, such as microfracturing the subchondral bone. Initially, microfracturing relieves symptoms; however, it results in fibrocartilaginous tissue in the long term.^4^ Fibrocartilage contains type I collagen which can buckle under high compression forces and eventually break, making it a less-durable repair tissue.^5-7^ Another treatment consists of using osteochondral autografts, which is suitable for small defects; however, for filling larger defects, the availability of the grafts becomes scarce.^4^ Alternatively, cell-based techniques like autologous chondrocyte implantation (ACI) from the first to fourth generation are frequently used. With these approaches, autologous chondrocytes are isolated from a biopsy taken from a non-load-bearing area of the cartilage.^3^ However, due to the low cell number in cartilage, a good quality biopsy is needed. This damages the healthy cartilage, and the chondrocytes must be further expanded to obtain enough cells for filling defects.^3,8^ During this 2D cell expansion, chondrocytes dedifferentiate resulting in fibroblastic cells which could lead to fibrocartilaginous repair tissue.^9^ Although, when these cells are subsequently grown in 3D matrices, re-differentiation can occur, leading to a hyaline cartilage tissue.^10^

A recent fourth-generation ACI method involves using a scaffold-free technique with cell spheroids to repair cartilage.^11^ These spheroids are 3-dimensional (3D) cell structures made through the static aggregation of single cells *in vitro* in low-attachment plates.^12^ Compared to 2-dimensional (2D) cultures, the benefits of using a 3D culture include improved cell viability and some matrix production.^13^ Moreover, 3D structures allow for cell-cell and cell-ECM contact, contributing to the improved preservation of the cells’ cartilaginous phenotype.^14^ An example of this approach is Spherox (previously known as chondrosphere), a cartilage repair treatment approved by the European Medicines Agency since 2017.^15^ The Spherox treatment has shown clinical improvements in patients with cartilage defects.^16^ However, the method still relies on obtaining autologous chondrocytes from healthy cartilage, which can cause donor-site morbidity, is time-consuming and costly, and can lead to dedifferentiated chondrocytes.^8,17^ Therefore, there is potential for improving both the scalability and quality of the cartilage spheroids.

To simplify and upscale the production of spheroids, several types of bioreactors that create a dynamic environment using media flow have been designed, such as a spinner flask.^18^ Crispim and Ito (2021) developed a protocol to make cartilage organoids with chondrocytes in a spinner flask using a matrix supplement called notochordal cell-derived matrix (NCM).^19^ Organoids are functional and complex mini-tissues, commonly formed by stem cells or primary progenitor cells, which self-assemble in 3D culture systems.^19,20^ NCM is primarily composed of type II collagen and GAGs, which are also the main components of cartilage ECM.^19^ It is derived from porcine intervertebral disks and has been shown to provide a matrix for chondrocytes to adhere to and self-assemble into matrix-rich cartilage organoids.^21,22^ In these organoids, chondrocytes were surrounded by a type VI collagen-rich PCM, and the ECM between cells was composed of GAGs and type II collagen.^19^ Furthermore, NCM promoted chondrocyte proliferation without dedifferentiation and preserved their chondrogenic phenotype.^19,21^ However, the major downside of using NCM includes its origin from a xenogeneic source and the limited knowledge of its components, restricting its clinical application. Furthermore, this type of organoid production is still constrained by the number of chondrocytes obtained from a biopsy, affecting scale-up possibilities.

Articular chondroprogenitor cells (ACPCs) could provide a solution to limit the biopsy size while ensuring enough cells for scalable organoid production. These cells are primarily located in the superficial layer of articular cartilage.^23^ ACPCs can be characterized by their adhesion to fibronectin and are positive for surface markers such as CD94e, CD90, CD105, CD166, Notch-1, STRO-1, and negative for CD45 and CD34.^24,25^ ACPCs can proliferate in 2D without losing their chondrogenic potential. Even when exceeding 30 passages, these cells retain the ability to produce hyaline cartilage ECM and maintain SOX9 protein levels.^26^ ACPCs have been shown to be more effective in producing a hyaline-like cartilage matrix when cultured in 3D after 2D expansion than chondrocytes.^27^ ACPCs have been used for cartilage repair strategies and showed type II collagen and proteoglycan formation without type I and type X collagen synthesis.^28^ Compared to chondrocytes and mesenchymal stem cells, another commonly investigated cell source for cartilage tissue engineering, ACPCs show minimal hypertrophy.^27,29^ Therefore, ACPCs seem to be a promising cell source for cartilage tissue repair. There are several ways to achieve chondrogenic differentiation in these progenitor cells, such as a decreased oxygen tension, the use of growth factors, and using inhibitor molecules.^30^ Multiple studies have been performed investigating the effect of bone morphogenetic proteins (BMPs) on chondrogenesis.^31-34^ BMPs promote chondrogenic differentiation in multiple ways during development, such as ensuring cell-cell contact during mesenchymal stem cell condensation in the limb bud and maintaining SOX9 expression.^31^ Specifically, BMP-9 was shown to have beneficial effects on the chondrogenic differentiation of ACPCs.^32-34^

The aim of this study was to create cartilage organoids with human primary ACPCs (hACPCs) in spinner flasks with BMP-9 to improve the scalability and quality of cartilage tissue engineering. Furthermore, the ability of these hACPC organoids to fuse to a larger neo-hyaline cartilage tissue was investigated.

## Materials and Methods

### hPAC and hACPC Isolation

The hACPCs and human primary articular chondrocytes (hPACs) were harvested from redundant articular cartilage tissue of anonymous patients undergoing total knee replacement surgery at Máxima Medical Center. The usage of redundant tissue was not subjected to Medical Research Involving Human Subjects Act and approved by the Local Research Committee (Medical Review Ethics Committee Máxima MC, no. N16.148). The hACPCs were obtained from 4 subjects (1 male and 3 females, 69 ± 11 years old), and the hPACs were obtained from 7 subjects (5 males and 2 females, 69 ± 9 years old). The cells were isolated from the cartilage by enzymatic digestion as previously described.^35^ The hACPCs were isolated from this cell suspension using a fibronectin adherence culture as previously described^27^ and passaged until passage 4 with hACPC expansion medium (high glucose Dulbecco’s modified Eagle medium (hgDMEM, 31966; Gibco, The Netherlands), 10% fetal bovine serum (FBS; Gibco), 1% penicillin/streptomycin (P/S; Lonza, Switzerland), 0.2 mM L-ascorbic acid 2-phosphate (AA, A8960; Sigma-Aldrich, The Netherlands), 1% MEM nonessential amino acids solution (NEAA, 11140050; Gibco), and 5 ng/ml bovine fibroblast growth factor (bFGF,233-FB; Peprotech, UK)) at 37°C and 5% CO_2_. To obtain passage 1 hPACs, cells after digestion were expanded using hPAC expansion medium (hgDMEM (31966), 10% FBS, and 1% P/S) at 37°C, and 5% CO_2_.

### hPAC and hACPC Organoid Culture

To produce hPAC organoids, the protocol described by Crispim and Ito (2021) was used.^19^ In short, 0.25 mg/ml NCM was dissolved in spinner flask medium (hgDMEM (419660), 5% FBS, 1% Insulin/transferrin/selenium-plus (ITS+ premix, 354352; Corning, The Netherlands), 1% P/S, 10 mM 4-(2-hydroxyethyl)-1-piperazineethanesulfonic acid (HEPES, 15630080; Gibco), 0.2 mM AA, and 1% NEAA) using an Ultra-Turrax T10 (IKA, Germany). Spinner flask cultures per donor with 50,000 P1 hPACs per ml medium were prepared (n = 7 donors). The spinner flasks (Wheaton, USA) were then kept on a magnetic stirring plate (Variomag Biosytem 4; Thermo Fisher Scientific, USA) at 60 r/min using the Biomodul 40B (Thermo Fischer Scientific) under hypoxic conditions (37°C, 5% CO_2_, and 2.5% O_2_) for 14 days with medium refreshments twice a week. During the medium changes, the NCM concentration was increased, initially to 0.5 mg/ml, followed by 1 mg/ml. To produce hACPC organoids, the same spinner flask medium without NCM was used and spinner flasks with 50,000 P4 hACPCs per ml medium were prepared (n = 4 donors). At the moment of cell seeding, no supplements were added to allow cell aggregation. During the first medium change on day 4, half of the medium was refreshed and on day 8 and 11, all medium was refreshed. During these medium changes, 100 ng/ml BMP-9 (120-07; Peprotech) was added to the spinner flask. Two representative samples (200 µl) from hACPC and hPAC spinner flask cultures were imaged with the EVOS XL Core microscope (AMEX1000; Life Technologies, USA) at each medium change to determine the size and number of organoids per culture. The size of the organoids was quantified by the Feret diameter using ImageJ software.

### Fusion

To evaluate the potential of the hPAC and hACPCs organoids to fuse into cartilage-like tissues, approximately 20 organoids were transferred to a low adhesive well (Round Bottom Ultra-Low Attachment 96-wells plate, 7007; Corning, The Netherlands) and spun down at 400 rcf for 10 minutes (n = 4 technical replicates for each of the 4 donors, totaling 16 replicates). Then, spinner flask medium, supplemented with 10 ng/ml transforming growth factor β1 (TGF-β1, 100-21; Peprotech) and 0.5 mM dexamethasone (D4902; Sigma-Aldrich), was added to the wells and cultured for 21 days at 37°C, 5% CO_2_, and 2.5% O_2_ with medium refreshments three times a week. The Feret diameter of the fused constructs was measured at day 21.

### Cell Tracking

To track how the cells from the organoids behave during fusion, a red and green fluorescent cell membrane labeling kit (PKH26 and PKH67, respectively; Sigma-Aldrich) were used to stain the cells after the spinner flask culture and before fusion using the protocol provided by the manufacturer. After staining, organoids with a red and green label were mixed and fused together as described above (n = 4 technical replicates for each of the 4 donors, totaling 16 replicates). After 21 days, optical sections were made with the TCS SP5 X confocal laser scanning microscope (Leica Microsystems, Germany). Due to a weak signal, the image brightness and contrast were increased digitally but similarly for all images.

### Mechanical Testing

To determine the mechanical properties of individual hPAC and hACPC organoids (n = 17 technical replicates for each of the 4 donors, totaling 68 replicates), an unconfined parallel plate compression test was performed using a micro-indenter (MicroTester G2; CellScale, Canada). The organoids were subjected to unconfined compression up to 50% of the initial diameter within 20 seconds with a 2 × 2 mm metal plate on the end of a cylindrical beam (Ø 0.2032 mm) in a container filled with phosphate-buffered saline (PBS; Sigma-Aldrich). Thereafter, the force-displacement curves between 0% and 20% deformation were obtained. Then, a fitting was performed to determine the Young’s Modulus using the nonlinear least squares fitting MATLAB tool together with the Extensive Theory described by Tatara^36^ and a previously described method by Kim *et al.*^37^ to calculate the radius of the contact area. After the compression tests, the organoids were stored at −20°C for biochemical analysis.

### Biochemical Analysis

Organoids after mechanical testing and halved fused constructs were lyophilized and digested in a papain digestion buffer (pH = 6.5; 100 mM phosphate buffer (Sigma-Aldrich), 5 mM L-cysteine (C-1276; Sigma-Aldrich), 5 mM ethylenediamine tetraacetic acid (EDTA, E4884; Sigma-Aldrich), and 130 µg/mL papain (P4762; Sigma-Aldrich)) for 16 hours at 60°C. To quantify sulfated GAG (sGAG) content, a 1-9-dimethyl methylene blue (DMMB, 341088, pH = 3.0; Sigma-Aldrich) assay was performed with chondroitin sulfate from shark cartilage (C4348; Sigma-Aldrich) as a reference.^38^ Then, the DNA content was measured with the Qubit dsDNA high-sensitivity kit (Q32851; Invitrogen, USA).

### Histology and Immunohistochemistry

To evaluate the cellular and matrix distribution in the organoids and halved fused constructs, samples were processed for histological and immunohistochemical analysis. The samples were fixed with formalin/eosin (3.7%/0.01%) (Z2902/HT110116; both Sigma-Aldrich) at 4°C overnight. Then, samples were removed and embedded in 3 w/v% sodium alginate (71238; Sigma-Aldrich). The samples were dehydrated in a Microm STP 120 Spin Tissue Processor (Thermo Fischer Scientific) followed by paraffin embedding (P3558; Sigma-Aldrich), and 5 µm sections were prepared. Histology was used to visualize the GAGs, where sections were first deparaffinized and rehydrated, followed by citrate buffer immersion (S1699; DAKO Agilent, USA) to remove the alginate. Then, the sections were stained with 1 w/v% alcian blue (A-5268; Sigma-Aldrich) and counterstained with Weigert’s Iron Hematoxylin (HT1079; Sigma-Aldrich). Finally, the samples were dehydrated, mounted with Entellan (1079610500; Sigma-Aldrich), and imaged with the Leica DMi8 microscope (Leica Microsystems).

For immunohistochemistry, sections were again deparaffinized and rehydrated. Antigen retrieval for type I and type II collagen staining was performed with pre-warmed 5 mg/mL hyaluronidase (H3506, Sigma-Aldrich) for 30 minutes at 37°C. Next, the samples were incubated with prewarmed 500 µg/mL pronase (53702; Millipore, USA) for 20 minutes at 37°C, followed by another 20 minutes at room temperature. Antigen retrieval for SOX9 and KI67 staining was done by immersing the samples in prewarmed citrate buffer at 96°C for 20 minutes followed by 30 minutes at room temperature. Antigen retrieval for type VI collagen was achieved by incubation with 0.5% pepsin in 10 mM hydrochloric acid (P7000 and 320331, respectively, both Sigma-Aldrich) at 37°C for 10 minutes. Samples stained for type I and II collagen were blocked with 5% normal goat serum (PCN5000, Gibco) in PBS for 45 minutes at room temperature. The sections for SOX9, KI67, and type VI collagen were blocked with 10% donkey serum (AB2337258; Jackson ImmunoResearch, UK) for 30 minutes at room temperature. After blocking, the samples were incubated with the primary antibodies diluted in PBS with 1% NGS or 1% donkey serum overnight (**Table 1**). Thereafter, the samples were incubated with the secondary antibody in PBS for 1 hour at room temperature (**Table 1**). Finally, the samples were stained with 1 µg/ml DAPI (D9542; Sigma-Aldrich) in PBS for 10 minutes to visualize cell nuclei, mounted with Mowiol (81381; Sigma-Aldrich), and imaged (Axio Observer 7; Zeiss, Germany).

**Table 1.** The Primary and Secondary Antibodies Used for Immunohistochemical Analysis and Their Concentration in PBS.

Detection of:	Primary antibody	Secondary antibody
Type I collagen	Mouse anti-collagen I (C2456; Sigma-Aldrich), 1:200	Goat anti-mouse 555 (A21424; Molecular Probes, USA), 1:200
Type II collagen	Mouse anti-collagen II (MA512789; Thermo Fisher Scientific), 1:200	Goat anti-mouse 555 (A21137; Molecular Probes Probes), 1:200
Type VI collagen	Rabbit anti-collagen VI (ab6588; Abcam), 1:300	Donkey anti-rabbit 488 (A21206; Invitrogen), 1:300
Ki67	Rabbit anti-Ki67 (rb1510-P0; Thermo Fisher Scientific), 1:200	Donkey anti-rabbit 647 (711-605-152; Jackson ImmunoResearch), 1:200
SOX9	Rabbit anti-SOX9 (PA-29537; Thermo Fisher Scientific), 1:14	Donkey anti-rabbit 647 (711-605-152; Jackson ImmunoResearch), 1:200

### Statistical Analysis

The results obtained from the size analysis, biochemical assays, and mechanical tests were analyzed using Prism 9 (GraphPad Software, USA; www.graphpad.com) to evaluate statistical differences between hACPC and hPAC organoids or fused constructs. First, the normality of the data was assessed using a Shapiro–Wilk’s test. To account for the different donors, nested *t*-tests were used with normally distributed data. If data were not normally distributed, the data were averaged per donor, and a nonparametric Mann–Whitney test was performed between the cell types. For all tests, *P* < 0.05 was considered significant.

## Results

### hACPCs with BMP-9 and hPACs with NCM Produced Cartilage Organoids

**Figure 1.**
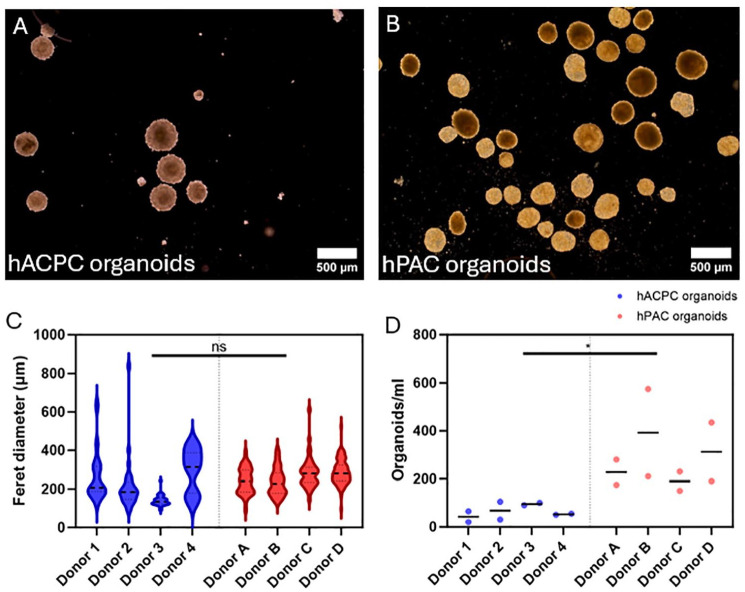
Macroscopic images of hACPC (A) and hPAC organoids (B) after a 14-day spinner flask culture. The size of the hACPC and hPAC organoids (C). Number of organoids per ml culture medium in the spinner flasks (D). * indicates *P* < 0.05 and ns *P* > 0.05.

Following a 14-day spinner flask culture, cartilage organoids were successfully formed by both hACPCs with BMP-9 stimulation and hPACs with NCM (**Fig. 1A** and **B**). The Feret diameter revealed no significant differences between the sizes of the hACPC and hPAC organoids (**Fig. 1C**, *P* = 0.69). However, a difference was seen in the quantity, with hPAC organoids exhibiting approximately a fourfold increase in numbers compared to hACPC organoids after the culture period, resulting in 281 ± 90 hPAC organoids and 61 ± 23 hACPC organoids per ml SF culture (**Fig. 1D**, *P* = 0.029).

**Figure 2.**
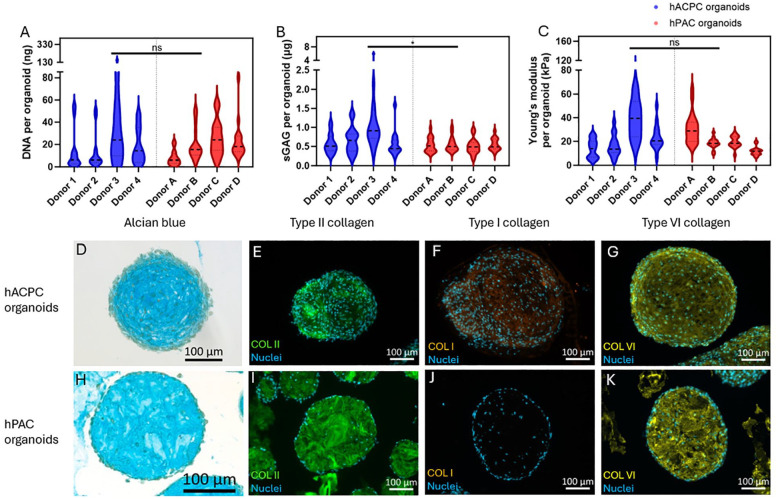
Characterization of the matrix in the cartilage organoids. The DNA (A) and sGAG (B) content per organoid and the Young’s modulus (C) of the hACPC and hPAC organoids after a 14-day spinner flask culture. Representative images of the hACPC and hPAC organoids stained for GAGs with alcian blue (D, H), for type II collagen (E, I), for type I collagen (F, J), and type VI collagen (G, K) with the nuclei stained with DAPI. * indicates *P* < 0.05 and ns *P* > 0.05.

Similar to the size, no significant differences were found between the organoids in terms of DNA content per organoid (**Fig. 2A**, *P* = 0.89). Interestingly, the sGAG content per organoid was significantly higher for the hACPC organoids compared to the hPAC organoids (0.82 ± 0.82 µg and 0.53 ± 0.19 µg sGAGs, respectively, *P* = 0.029). In addition, the mechanical properties were not significantly different, where the Young’s modulus of the hACPC organoids was 24.0 ± 17.9 kPa, and for the hPAC organoids, it was 20.8 ± 10.7 kPa (**Fig. 2C**, *P* = 0.89). The alcian blue stain supported the biochemical assay by showing the presence of GAGs on both organoids (**Fig. 2D** and **H**). Hematoxylin and DAPI revealed cellular distribution, with hACPC organoids displaying a more uniform distribution compared to hPAC organoids, where the hPACs predominantly lined the outer periphery (**Fig. 2D-K**). Both organoid types exhibited types II and VI collagen throughout the whole structure (**Fig. 2E, I, G**, and **K**). Type VI collagen shows cavities around the cells in hACPC organoids, in contrast to the hPAC organoids (**Fig. 2G** and **K**). However, some type I collagen was present in hACPC organoids, which was not observed in hPAC organoids (**Fig. 2F** and **J**).

### Fusing hACPC Organoids Created a Neo-Hyaline Cartilage Construct with Uniformly Distributed Matrix

**Figure 3.**
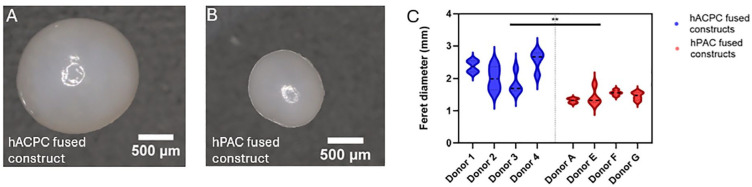
Macroscopic images of the hACPC (A) and hPAC (B) fused constructs after allowing ±20 organoids to fuse for 21 days in low-adherent wells. The size of the hACPC- and hPAC-fused constructs (C). ** indicates *P* < 0.01.

**Figure 4.**
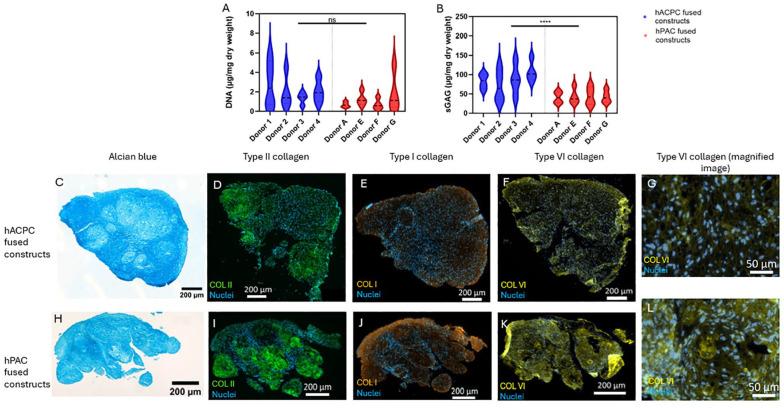
Characterization of the matrix in the fused constructs. The DNA (A) and GAG (B) content per dry weight of the fused constructs after a 21-day static culture. Representative images of the hACPC- and hPAC-fused constructs stained for GAGs with alcian blue (C, H), for type II collagen (D, I), for type I collagen (E, J), and type VI collagen (F, K) with the nuclei stained with DAPI. Magnified images of the hACPC- and hPAC-fused constructs stained for type VI collagen and with DAPI (G, L). Please note the different-sized scale bars, as the microscopy images of the fused constructs needed tile scans. *** indicates *P* < 0.001 and ns *P* > 0.05.

Both organoid types successfully fused, forming a white, unified spheroid (**Fig. 3A** and **B**). After 21 days, the hACPC fused constructs were 1.5-fold larger than the hPAC constructs (**Fig. 3C**, *P* = 0.0051). While the DNA per dry weight did not differ between cell types (**Fig. 4A**, *P* = 0.064), the sGAG content per dry weight did show significantly higher values for the hACPC constructs (**Fig. 4B**, *P* < 0.0001). Specifically, hACPC constructs contained almost twice the amount of sGAGs compared to the hPAC constructs (87.7 ± 32.9 µg sGAGs and 43.0 ± 17.3 µg sGAGs per mg dry weight, respectively), which correlates to the alcian blue stains (**Fig. 4C** and **H**). The histochemical and immunohistochemical images of the constructs revealed distinct matrix distribution patterns. Type II and type VI collagen were uniformly distributed throughout the hACPC fused constructs (**Fig. 4D**). In contrast, type II collagen in hPAC constructs appeared to be concentrated in the original organoids rather than in the fusion areas (**Fig. 4I**). Both constructs showed type VI collagen in the pericellular region (**Fig. G** and **L**); however, there was overall more type VI collagen in the hPAC-fused constructs than in the hACPCs constructs (**Fig. 4F** and **K**). Interestingly, type VI collagen was also present in the fusion regions of hPAC constructs, in contrast to type II collagen (**Fig. 4I, K**). The distribution of cells mirrored the matrix distribution trends, with cells evenly distributed throughout the hACPC constructs and less uniformly distributed in the hPAC constructs (**Fig. 4D-F** and **4I-K**). In the hPAC constructs, there were primarily cells in the fused regions. Type I collagen was seen for both constructs mainly at the outer periphery of the fusion masses (**Fig. 4E** and **J**).

### hACPC Migrated Throughout the Fused Constructs

**Figure 5.**
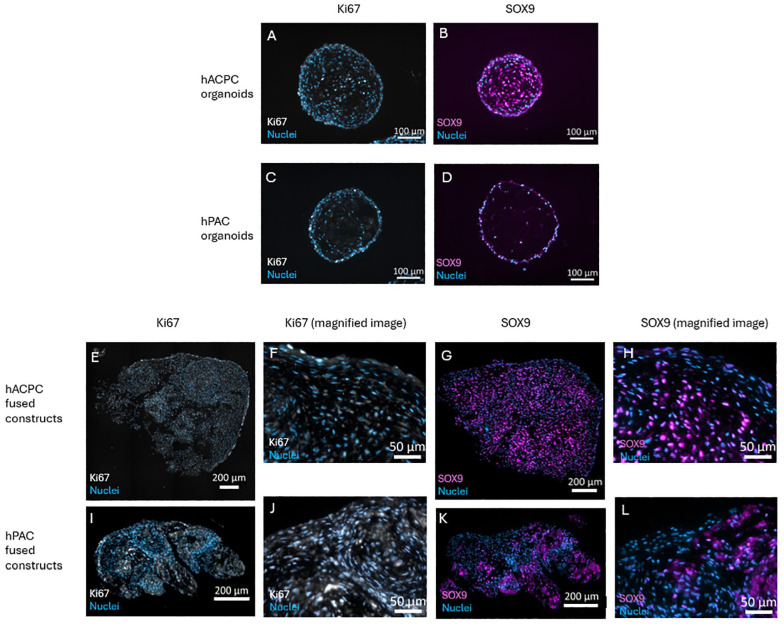
Characterization of the cells in the organoids and fused constructs. Representative images of the hACPC and hPAC organoids stained for Ki67 (A, C) and SOX9 (B, D) with the nuclei stained with DAPI. Representative images of the hACPC- and hPAC-fused constructs stained for Ki67 with the nuclei stained with DAPI (E, I) with zoomed images (F, J). Representative images of the hACPC- and hPAC-fused constructs stained for SOX9 with the nuclei stained with DAPI (G, K) with zoomed images (H, L). Please note the different-sized scale bars, as the microscopy images of the fused constructs needed tile scans.

In addition to the matrix, the cells within the organoids and fusion masses were evaluated. Both hPAC and hACPC organoids showed a modest Ki67 expression (**Fig. 5A** and **C**). SOX9 was expressed in both organoid types, where the hACPCs expressed SOX9 in the core as well as at the outer periphery of the organoids (**Fig. 5B** and **D**). For the fused constructs, the NCM seemed to result in some background staining for both markers (**Fig. 5I** and **K**). The Ki67 and SOX9 expression were more uniformly distributed in the hACPC constructs than hPAC constructs (**Fig. 5E, I, G**, and **K**). Magnified images revealed that the hACPC constructs contained more SOX9-expressing cells (**Fig. 5H** and **L**), while the hPACs in their fused construct expressed more Ki67 (**Fig. 5J** and **F**).

**Figure 6.**
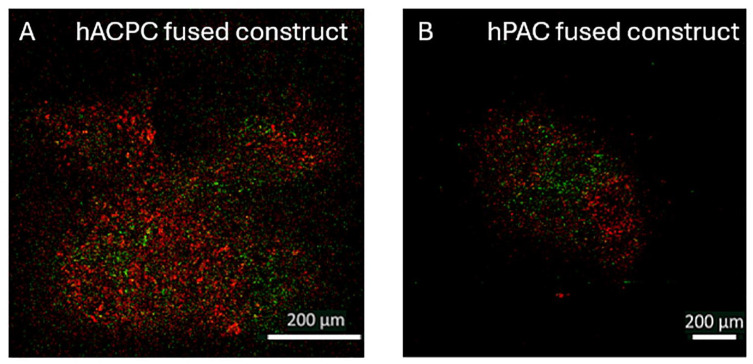
Cell position after organoid fusion in the hACPC (A) and hPAC-fused constructs (B) indicated with a red or green cell membrane labeling dye. Please note the different-sized scale bars, as the microscopy images of the fused constructs needed tile scans.

Following a 21-day fusion period, the green and red fluorescent cell membrane labels were either not detectable or faint in most samples. Therefore, the brightness and contrast were enhanced to evaluate the cellular distribution. In the hPAC-fused constructs, the cells remained close to their original organoid, with minimal invasion of red- or green-stained cells into adjacent organoids (**Fig. 6B**). In contrast, the hACPCs of neighboring organoids showed more mixing within their fused constructs (**Fig. 6A**).

## Discussion

In this study, the overall aim was to advance cartilage tissue repair by improving the scalability and quality of the engineered cartilage using cartilage organoids. It was shown that cartilage organoids can be produced by using a sustainable cell source, hACPCs, in spinner flasks, which was fast, needed only little labor, and was easily amenable to up-scaling. Even though some type I collagen was found in the hACPC organoids, the average Young’s modulus was not affected compared to hPAC organoids without type I collagen. Fusing the organoids together showed that using hACPCs with BMP-9 instead of hPACs with NCM, led to a neo-hyaline cartilage tissue with a uniform distribution of cells and matrix.

In contrast to hPACs, which required NCM to form aggregates, hACPCs exhibited self-aggregation in the spinner flasks during the first 4 days, forming cellular aggregates. Similarly, mesenchymal cells condense in the limb bud during development, followed by the production of cartilage matrix.^31^ The hACPC condensation prior to stimulation with a chondrogenic factor might be beneficial for the hACPC differentiation as it was shown that cell–cell contact is also crucial to start chondrogenic differentiation in the limb bud cells.^39^ Furthermore, this cell–cell contact might also be favorable for matrix production.^39^ Moreover, previous studies showed that hACPCs exceed hPACs in cartilage matrix production, besides proliferation.^30^ This correlates to this study where the aggregated hACPCs synthesized a substantial amount of matrix in a short culture period to compensate for the absence of NCM, which was added to the hPAC culture (0.82 ± 0.82 µg sGAG per hACPC organoid and 0.53 ± 0.19 µg sGAG per hPAC organoid). Interestingly, hACPC donor 3 had higher sGAG and DNA content per organoid as well as a higher Young’s modulus compared to the other hACPCs donors. Initially, organoids were manually selected by different investigators for mechanical testing followed by biochemical assays. This process could have introduced a bias as organoids selected for donor 3 were larger than the average, most likely resulting in higher biochemical content and mechanical properties. It is important to note that the current matrix analysis methods did not permit a direct comparison of matrix synthesis by hACPCs and hPACs in the organoids. The hPAC organoids were formed using a matrix additive rich in GAGs, type II collagen, and type VI collagen,^19,40^ making it challenging to distinguish whether the measured and visualized matrix in hPAC organoids was synthesized by the cells or originated from the NCM. It was assumed that most of the matrix in hPAC organoids is NCM because of the central distribution of the ECM in the hPAC organoids and the short spinner flask culture. Future experiments could try to distinguish cell-produced matrix from the supplemented NCM, using for example analog labeling, to assess the metabolic activity of the cells in the organoids, as it is hypothesized that metabolically active cartilage organoids may ensure better integration into native tissue.^21^

Adding BMP-9 to the hACPC aggregates led to similar-sized organoids and similar matrix content compared to the hPAC organoids, although approximately 4 times fewer organoids. This difference might arise from the need of the hACPCs to produce their own ECM, whereas the hPACs were provided with an ECM, and might be resolved by starting with a higher hACPC concentration in the spinner flasks. To specify the desired amount of hACPC organoids, future studies should investigate how many cartilage organoids per square centimeter are required to fill an articular cartilage defect. Multiple studies have shown the beneficial effects of BMP-9 on the maturation of ACPCs. Morgan *et al*.^32^ showed that pelleted ACPCs treated with BMP-9 exhibited a chondrogenic phenotype with GAG and type II collagen production. In addition, Padmaja *et al*.^34^ explored the effects of BMP-9 during 2D ACPC expansion and subsequent pellet culture under TGF-β1 stimulation. Their findings confirmed that BMP-9 activated chondrogenesis in ACPCs, particularly evident in the upregulation of Col2a1. TGF-β1 is another growth factor described as a chondrogenic stimulant for ACPCs, and it is known for increasing the cartilage matrix synthesis by chondrocytes.^32,41^ Therefore, BMP-9 was replaced by TGF-β1 with dexamethasone during fusion culture in this study, as further hACPC maturation and matrix production by hPACs and hACPC was desired. Interestingly, the hACPC organoids grew into larger constructs than the hPAC organoids, while the fusion started with a comparable number of similar-sized organoids. Furthermore, the concentration of sGAGs per dry weight in the hACPC constructs is closer to the 15% sGAGs per dry weight found in adult human articular cartilage, compared to the hPAC constructs (87.7 ± 32.9 µg (8.77%) and 43.0 ± 17.3 µg (4.30%) sGAGs per mg dry weight, respectively).^42^ This study also showed a fast fusion of the organoids, as a spherical construct for both cell types was already observed after 3 days. A study of the commercially approved Spherox system by Anderer and Liber^43^ proved the fusion of their aggregates in low adhesive agarose-coated wells plates using autologous serum, resulting in the formation of a uniform spherical construct after 8 days.

In this study, hACPCs derived from osteoarthritic cartilage tissue were used. In previous research, osteoarthritic cartilage contained significantly fewer hACPCs, which were slower in proliferation and produced less cartilage matrix compared to hACPCs derived from healthy tissue.^30,44^ In contrast, others report faster proliferation of hACPCs and increased matrix production in severe osteoarthritis cartilage samples compared to healthy cartilage tissue.^30,45^ Similarly, in this study, a substantial number of matrix-producing hACPCs were successfully retrieved from osteoarthritic cartilage by selection using a fibronectin adherence culture and by expanding up to passage 4. As described before, hACPCs can be expanded up to 30 passages without losing their chondrogenic properties.^27^ The possibility for substantial proliferation while retaining their chondrogenicity distinguishes the osteoarthritic hACPCs from the osteoarthritic hPACs,^27^ indicating that osteoarthritic patients might benefit from autologous hACPC-based cell therapy. Nevertheless, future studies should include hACPCs from healthy as well as juvenile sources to address the contradictions in literature and evaluate how the osteoarthritic environment impacts the quality and scalability of the hACPCs.

It is important to note that type I collagen was found in the hACPC organoids. Even though ACPCs are known for keeping their chondrogenic potential upon expansion, several studies reported type I collagen in their ACPC-derived constructs.^33,46,47^ Possibly, the osteoarthritic environment in which the hACPCs were residing before isolation could have been the cause of their type I collagen production.^27^ Future studies should therefore quantify the type II and type I collagen, as a high type II/I collagen ratio is a marker for chondrogenicity of the cells.^48^ The absence of type I collagen in the hPAC organoids could be attributed to either the lack of dedifferentiated hPACs, or to the possibility that the hPACs did not produce any matrix at all. This uncertainty arises as the current analysis methods did not allow to determine whether the hPACs in the organoids with NCM produced matrix. Following the fusion culture, type I collagen was observed in both hACPC as hPAC constructs at the outer periphery of the construct. A previous study of Spherox similarly identified type I collagen in the outer periphery of their fusion masses in a low-adherent system when cultured with FBS.^43^ This observation may be attributed to the low-adherent culture system, generating higher tensions at the outer rim of the construct, thereby stimulating type I collagen synthesis. Future research should investigate whether there could be a decrease in type I collagen synthesis using adherent culture systems, given that these engineered cartilage constructs are envisioned for filling adherent osteochondral defects. As some type I collagen in the hACPC organoids was observed, the Young’s modulus of the hACPC and hPAC organoids was measured. Fibrocartilaginous tissue, which contains type I collagen, has inferior mechanical properties compared to hyaline-like cartilage.^7^ Fortunately, no differences were found between the Young’s moduli of the hACPC and hPAC organoids (24.0 ± 17.9 and 20.8 ± 10.7 kPa, respectively). The Hertzian half-space contact mechanics model is a commonly used model to evaluate mechanical properties; however, it is limited to small deformations. Therefore, the Extensive Theory, as introduced by Tatara,^36^ was used to define the Young’s modulus of the organoids. Using this theory, the bulk properties from spheres undergoing larger deformations can be obtained, which were shown to be more accurate than using the Hertz model with up to 20% compression.^36^ To calculate the radius of the contact area, a geometric calculation by Kim *et al*.^37^ was included. A compression test conducted by Omelyanenko *et al*. (2018)^49^ revealed a modulus of 9 kPa for their chondrospheres after 14 days, while this study shows an average Young’s modulus of 22.3 ± 14.5 kPa.

Type II collagen was produced by the hACPCs in the fused construct during a 21-day culture period, as it was uniformly observed throughout the construct, which again shows the fast production of ECM by the hACPCs. Contrary, it seems that the hPACs produced almost no type II collagen during fusion, as the type II collagen is predominantly located in the original organoids rather than in the fused areas. Even though type VI collagen was present in the PCM throughout both fused constructs, hPAC constructs also seemed to have type VI collagen in the ECM, in contrast to the hACPC construct. This could indicate that the tissue derived from hACPCs might not be as mature as the tissue made by hPACs, as previous studies similarly demonstrated weak type VI collagen staining in immature cartilage tissue compared to a more mature tissue.^40^ Contrary, an increased type VI collagen production by the hPACs may also indicate a pathological state, as multiple studies showed increased amounts of type VI collagen in osteoarthritic cartilage.^50^

Besides matrix visualization, the cell membrane of the cells within the organoids were stained with a green or red fluorescent dye to evaluate the migration of the hACPCs and hPACs during fusion.^51^ It was found that while the hACPCs seemed to migrate and invade neighboring organoids, the hPACs merely migrated into fusion areas. Anderer and Libera (2002) observed a similar behavior where they describe that the chondrocytes from their spheroids migrated over cartilage explants rather than invading them.^43^ The lack of cell invasion in hPAC constructs may result in weaker tissues, which is supported by the tears in the hPAC construct histology sections. This could suggest a weaker connection between the hPAC organoids in their fused construct compared to the hACPC construct. After fusion culture, most of the signal from the cell membrane label was lost, mostly in the hACPC fused constructs. This could indicate more cell proliferation in the hACPC constructs, as the intensity of a cell membrane dye decreases when cells proliferate. The proliferation and migration into neighboring organoids by the hACPCs, which were derived from osteoarthritic tissue, is also commonly seen with cartilage injuries or osteoarthritis, where the progenitor cells seem to proliferate and migrate toward the damaged zones.^52^ Since both matrix production and proliferation were observed in the hACPC constructs, future studies should investigate whether multiple cell subsets are present or if the hACPCs were capable of both ECM synthesis and proliferation.

Staining for Ki67, indicating proliferation,^53^ and SOX9, indicating chondrogenicity,^54^ showed variation between samples and donors for the fused constructs. Overall, most hACPCs in the fused constructs were SOX9-positive, which is consistent with previous research.^54^ These constructs showed type I collagen near the periphery, where the cells did not express SOX9, indicating the lack of a chondrogenic phenotype at the edges. Contrary, hACPC organoids showed SOX9 expression throughout the whole organoid, while type I collagen was also seen in most of the hACPC organoids. Although substantial hACPC proliferation in the fused construct was assumed based on the lack of signal from the cell membrane label, Ki67 expression was not notably high. Interestingly, only a few SOX9-positive cells were seen in the hPAC-fused construct, which may indicate hypertrophy as chondrocytes loose their SOX9 expression at this stage.^54^ This was supported by the substantial number of Ki67-expressing hPACs in the fused areas of the constructs, and proliferative chondrocytes tend to be dedifferentiated or hypertrophic cells,^55^ which is not beneficial for the quality of engineered cartilage. Finally, long-term *ex vivo* studies could be performed using the hACPC organoids in a cartilage repair model, such as osteochondral plugs, to asses the repair potential and move forward to advancing cartilage repair strategies.

## Conclusion

This study demonstrated that hACPCs were able to self-assemble into cartilage organoids, and grow and produce a cartilage-like matrix with BMP-9 supplementation in spinner flasks. Organoids created with hACPCs and BMP-9 resembled cartilage organoids made with hPACs and NCM. The hACPC organoids and their fused constructs contained a uniformly distributed matrix rich in sGAGs and type II collagen. They also resulted in larger fused constructs than hPAC constructs. However, some differences between the organoids created from the two cell types were encountered, such as the presence of type I collagen in hACPC organoids. Nevertheless, this type I collagen did not affect the Young’s modulus, nor was there a visible accumulation of type I collagen in the hACPC-fused constructs. Using hACPCs, which can proliferate without dedifferentiating, combined with a technique facilitating the rapid and scalable production of cartilage organoids, holds the potential to enhance the clinical applicability of cartilage organoids in cartilage repair strategies.

## References

[1] Sophia Fox AJ Bedi A Rodeo SA . The basic science of articular cartilage: structure, composition, and function. Sports Health. 2009;1(6):461-8. 10.1177/1941738109350438 PMC3445147 23015907

[2] Wilusz RE Sanchez-Adams J Guilak F . The structure and function of the pericellular matrix of articular cartilage. Matrix Biol. 2014;3925-32. 10.1016/j.matbio.2014.08.009 PMC4198577 25172825

[3] Oldershaw RA . Cell sources for the regeneration of articular cartilage: the past, the horizon and the future. Int J Exp Pathol. 2012;93(6):389-400. 23075006 10.1111/j.1365-2613.2012.00837.x PMC3521894

[4] Marcacci M Filardo G Kon E . Treatment of cartilage lesions: what works and why. Injury. 2013;44(Suppl 1):S11-5. 10.1016/S0020-1383(13)70004-4 23351863

[5] Freemont AJ Hoyland J . Lineage plasticity and cell biology of fibrocartilage and hyaline cartilage: its significance in cartilage repair and replacement. Eur J Radiol. 2006;57(1):32-6. 10.1016/j.ejrad.2005.08.008 16182502

[6] Bielajew BJ Hu JC Athanasiou KA . Collagen: quantification, biomechanics and role of minor subtypes in cartilage. Nat Rev Mater. 2020;5(10):730-47. 10.1038/s41578-020-0213-1 PMC8114887 33996147

[7] Hunziker EB . Articular cartilage repair: basic science and clinical progress: a review of the current status and prospects. Osteoarthritis Cartilage. 2002;10(6):432-63. 10.1053/joca.2002.0801 12056848

[8] Niemeyer P Laute V John T Becher C Diehl P Kolombe T , et al. The effect of cell dose on the early magnetic resonance morphological outcomes of autologous cell implantation for articular cartilage defects in the knee. Am J Sports Med. 2016;44(8):2005-14. 10.1177/0363546516646092 27206690

[9] Kisiday JD . Expansion of chondrocytes for cartilage tissue engineering: a review of chondrocyte dedifferentiation and redifferentiation as a function of growth in expansion culture. Regen Med Front. 2020;2(1):e200002.

[10] Benya PD Shaffer JD . Dedifferentiated chondrocytes reexpress the differentiated collagen phenotype when cultured in agarose gels. Cell. 1982;30(1):215-24. 10.1016/0092-8674(82)90027-7 7127471

[11] Armoiry X Cummins E Connock M Metcalfe A Royle P Johnston R , et al. Autologous chondrocyte implantation with chondrosphere for treating articular cartilage defects in the knee: an evidence review group perspective of a NICE single technology appraisal. Pharmacoeconomics. 2019;37(7):879-86. 10.1007/s40273-018-0737-z 30426462

[12] Eschen C Kaps C Widuchowski W Fickert S Zinser W Niemeyer P , et al. Clinical outcome is significantly better with spheroid-based autologous chondrocyte implantation manufactured with more stringent cell culture criteria. Osteoarthr Cartil Open. 2020;2(1):100033. 36474562 10.1016/j.ocarto.2020.100033 PMC9718150

[13] Laschke MW Menger MD . Life is 3D: boosting spheroid function for tissue engineering. Trends Biotechnol. 2017;35(2):133-44. 10.1016/j.tibtech.2016.08.004 27634310

[14] He H He Q Xu F Zhou Y Ye Z Tan WS . Dynamic formation of cellular aggregates of chondrocytes and mesenchymal stem cells in spinner flask. Cell Prolif. 2019;52(4):e12587. 10.1111/cpr.12587 PMC6669002 31206838

[15] Spherox European Medicines Agency. Accessed November 14, 2023. https://www.ema.europa.eu/en/medicines/human/EPAR/spherox.

[16] Niemeyer P Laute V Zinser W John T Becher C Diehl P , et al. Safety and efficacy of matrix-associated autologous chondrocyte implantation with spheroid technology is independent of spheroid dose after 4 years. Knee Surg Sports Traumatol Arthrosc. 2020;28(4):1130-43. 10.1007/s00167-019-05786-8 31897548

[17] Kaps C Roël G Eschen C . Method for cultivation of cartilage and spheroids thereof. Published Online 2022 [Accessed November 14, 2023]. https://patents.google.com/patent/US20220220443A1/en?oq=US+2022%2f0220443+A1.

[18] Fu L Li P Li H Gao C Yang Z Zhao T , et al. The application of bioreactors for cartilage tissue engineering: advances, limitations, and future perspectives. Stem Cells Int. 2021;2021:6621806. 33542736 10.1155/2021/6621806 PMC7843191

[19] Crispim JF Ito K . De novo neo-hyaline-cartilage from bovine organoids in viscoelastic hydrogels. Acta Biomater. 2021;128236-49. 10.1016/j.actbio.2021.04.008 33894352

[20] Kratochvil MJ Seymour AJ Li TL Paşca SP Kuo CJ Heilshorn SC . Engineered materials for organoid systems. Nat Rev Mater. 2019;4(9):606-22. 10.1038/s41578-019-0129-9 PMC7864216 33552558

[21] Kleuskens MWA Crispim JF van Donkelaar CC Janssen RPA Ito K . Evaluating initial integration of cell-based chondrogenic constructs in human osteochondral explants. Tissue Eng Part C Methods. 2022;28(1):34-44. 35018813 10.1089/ten.TEC.2021.0196

[22] de Vries S Doeselaar MV Meij B Tryfonidou M Ito K . Notochordal cell matrix as a therapeutic agent for intervertebral disc regeneration. Tissue Eng Part A. 2019;25(11-12):830-41. 10.1089/ten.TEA.2018.0026 29739272

[23] Dowthwaite GP Bishop JC Redman SN Khan IM Rooney P Evans DJR , et al. The surface of articular cartilage contains a progenitor cell population. J Cell Sci. 2004;117(6):889-97. 10.1242/jcs.00912 14762107

[24] Fellows CR Matta C Zakany R Khan IM Mobasheri A . Adipose, bone marrow and synovial joint-derived mesenchymal stem cells for cartilage repair. Front Genet. 2016;7:213. 28066501 10.3389/fgene.2016.00213 PMC5167763

[25] Vinod E Kachroo U Amirtham SM Ramasamy B Sathishkumar S . Comparative analysis of fresh chondrocytes, cultured chondrocytes and chondroprogenitors derived from human articular cartilage. Acta Histochem. 2020;122(1):151462. 31733827 10.1016/j.acthis.2019.151462

[26] Khan IM Bishop JC Gilbert S Archer CW . Clonal chondroprogenitors maintain telomerase activity and Sox9 expression during extended monolayer culture and retain chondrogenic potential. Osteoarthritis Cartilage. 2009;17(4):518-28. 10.1016/j.joca.2008.08.002 19010695

[27] Rikkers M Korpershoek JV Levato R Malda J Vonk LA . Progenitor cells in healthy and osteoarthritic human cartilage have extensive culture expansion capacity while retaining chondrogenic properties. Cartilage. 2021;13(2_Suppl):129S-42. 10.1177/19476035211059600 PMC8804833 34802263

[28] Jiang Y Cai Y Zhang W Yin Z Hu C Tong T , et al. Human cartilage-derived progenitor cells from committed chondrocytes for efficient cartilage repair and regeneration. Stem Cells Transl Med. 2016;5(6):733-44. 10.5966/sctm.2015-0192 PMC4878331 27130221

[29] Vinod E Parameswaran R Ramasamy B Kachroo U . Pondering the potential of hyaline cartilage–derived chondroprogenitors for tissue regeneration: a systematic review. Cartilage. 2021;13(2_Suppl):34S-52. 10.1177/1947603520951631 PMC8804774 32840123

[30] Rikkers M Korpershoek JV Levato R Malda J Vonk LA . The clinical potential of articular cartilage-derived progenitor cells: a systematic review. NPJ Regen Med. 2022;7(1):2. 10.1038/s41536-021-00203-6 PMC8748760 35013329

[31] Yoon BS Lyons KM . Multiple functions of BMPs in chondrogenesis. J Cell Biochem. 2004;93(1):93-103. 15352166 10.1002/jcb.20211

[32] Morgan BJ Bauza-Mayol G Gardner OFW Zhang Y Levato R Archer CW , et al. Bone morphogenetic protein-9 is a potent chondrogenic and morphogenic factor for articular cartilage chondroprogenitors. Stem Cells Dev. 2020;29(14):882-94. 10.1089/scd.2019.0209 PMC7374587 32364057

[33] Gardner OFW Zhang Y Khan IM . BMP9 is a potent inducer of chondrogenesis, volumetric expansion and collagen type II accumulation in bovine auricular cartilage chondroprogenitors. PLoS ONE. 2023;18(11):e0294761. 10.1371/journal.pone.0294761 PMC10664884 37992123

[34] Padmaja K Amirtham SM Rebekah G Sathishkumar S Vinod E . Supplementation of articular cartilage-derived chondroprogenitors with bone morphogenic protein-9 enhances chondrogenesis without affecting hypertrophy. Biotechnol Lett. 2022;44(9):1037-49. 10.1007/s10529-022-03280-9 35920961

[35] Kleuskens MWA Crispim JF van Doeselaar M van Donkelaar CC Janssen RPA Ito K . Neo-cartilage formation using human nondegenerate versus osteoarthritic chondrocyte-derived cartilage organoids in a viscoelastic hydrogel. J Orthop Res. 2023;41(9):1902-15. 10.1002/jor.25540 36866819

[36] Tatara Y . Large deformations of a rubber sphere under diametral compression: part 1: theoretical analysis of press approach, contact radius and lateral extension. JSME Int Journal Ser A, Mech Mater Eng. 1993;36(2):190-6.

[37] Kim K Cheng J Liu Q Wu XY Sun Y . Investigation of mechanical properties of soft hydrogel microcapsules in relation to protein delivery using a MEMS force sensor. J Biomed Mater Res A. 2010;92(1):103-13. 10.1002/jbm.a.32338 19165782

[38] Farndale RW Sayers CA Barrett AJ . A direct spectrophotometric microassay for sulfated glycosaminoglycans in cartilage cultures. Connect Tissue Res. 1982;9(4):247-8. 10.3109/03008208209160269 6215207

[39] Zhang W Green C Stott NS . Bone morphogenetic protein-2 modulation of chondrogenic differentiation in vitro involves gap junction-mediated intercellular communication. J Cell Physiol. 2002;193(2):233-43. 10.1002/jcp.10168 12385001

[40] Watanabe K Mochida J Nomura T Okuma M Sakabe K Seiki K . Effect of reinsertion of activated nucleus pulposus on disc degeneration: an experimental study on various types of collagen in degenerative discs. Connect Tissue Res. 2003;44(2):104-8. 12745677

[41] Wu M Wu S Chen W Li YP . The roles and regulatory mechanisms of TGF-β and BMP signaling in bone and cartilage development, homeostasis, and disease. Cell Res. 2024;34(2):101-23. 10.1038/s41422-023-00918-9 PMC10837209 38267638

[42] Elliott RJ Gardner DL . Changes with age in the glycosaminoglycans of human articular cartilage. Ann Rheum Dis. 1979;38(4):371-7. 10.1136/ard.38.4.371 PMC1000374 496451

[43] Anderer U Libera J . In vitro engineering of human autogenous cartilage. J Bone Miner Res. 2002;17(8):1420-9. 10.1359/jbmr.2002.17.8.1420 12162496

[44] Xia Z Ma P Wu N Su X Chen J Jiang C , et al. Altered function in cartilage derived mesenchymal stem cell leads to OA-related cartilage erosion. Am J Transl Res. 2016;8(2):433-46. PMC4846894 27158337

[45] Hoshiyama Y Otsuki S Oda S Kurokawa Y Nakajima M Jotoku T , et al. Chondrocyte clusters adjacent to sites of cartilage degeneration have characteristics of progenitor cells. J Orthop Res. 2015;33(4):548-55. 10.1002/jor.22782 PMC4454425 25691232

[46] Mancini IAD Schmidt S Brommer H Pouran B Schäfer S Tessmar J , et al. A composite hydrogel-3D printed thermoplast osteochondral anchor as example for a zonal approach to cartilage repair: in vivo performance in a long-term equine model. Biofabrication. 2020;12(3):035028. 32434160 10.1088/1758-5090/ab94ce

[47] Schmidt S Abinzano F Mensinga A Teßmar J Groll J Malda J , et al. Differential production of cartilage ECM in 3D agarose constructs by equine articular cartilage progenitor cells and mesenchymal stromal cells. Int J Mol Sci. 2020;21(19):7071. 32992847 10.3390/ijms21197071 PMC7582568

[48] Woodfield TB Miot S Martin I van Blitterswijk CA Riesle J . The regulation of expanded human nasal chondrocyte re-differentiation capacity by substrate composition and gas plasma surface modification. Biomaterials. 2006;27(7):1043-53. 10.1016/j.biomaterials.2005.07.032 16125219

[49] Omelyanenko NP Karalkin PA Bulanova EA Koudan EV Parfenov VA Rodionov SA , et al. Extracellular matrix determines biomechanical properties of chondrospheres during their maturation in vitro. Cartilage. 2020;11(4):521-31. 10.1177/1947603518798890 PMC7488948 30221989

[50] Horikawa O Nakajima H Kikuchi T Ichimura S Yamada H Fujikawa K , et al. Distribution of type VI collagen in chondrocyte microenvironment: study of chondrons isolated from human normal and degenerative articular cartilage and cultured chondrocytes. J Orthop Sci. 2004;9(1):29-36. 14767702 10.1007/s00776-003-0737-4

[51] Khawar IA Park JK Jung ES Lee MA Chang S Kuh HJ . Three dimensional mixed-cell spheroids mimic stroma-mediated chemoresistance and invasive migration in hepatocellular carcinoma. Neoplasia. 2018;20(8):800-12. 10.1016/j.neo.2018.05.008 PMC6034588 29981501

[52] Jiang Y Tuan RS . Origin and function of cartilage stem/progenitor cells in osteoarthritis. Nat Rev Rheumatol. 2015;11(4):206-12. 10.1038/nrrheum.2014.200 PMC5413931 25536487

[53] Scholzen T Gerdes J . The Ki-67 protein: from the known and the unknown. J Cell Physiol. 2000;182(3):311-22. 10.1002/(SICI)1097-4652(200003)182:3<311::AID-JCP1>3.0.CO;2-9 10653597

[54] de Crombrugghe B Lefebvre V Behringer RR Bi W Murakami S Huang W . Transcriptional mechanisms of chondrocyte differentiation. Matrix Biol. 2000;19(5):389-94. 10.1016/s0945-053x(00)00094-9 10980415

[55] Charlier E Deroyer C Ciregia F Malaise O Neuville S Plener Z , et al. Chondrocyte dedifferentiation and osteoarthritis (OA). Biochem Pharmacol. 2019;16549-65. 10.1016/j.bcp.2019.02.036 30853397

